# *In Vivo* Deletion of the *Cebpa* +37 kb Enhancer Markedly Reduces *Cebpa* mRNA in Myeloid Progenitors but Not in Non-Hematopoietic Tissues to Impair Granulopoiesis

**DOI:** 10.1371/journal.pone.0150809

**Published:** 2016-03-03

**Authors:** Hong Guo, Stacy Cooper, Alan D. Friedman

**Affiliations:** Division of Pediatric Oncology, Johns Hopkins University School of Medicine, Baltimore, Maryland, United States of America; Kanazawa University, JAPAN

## Abstract

The murine *Cebpa* gene contains a +37 kb, evolutionarily conserved 440 bp enhancer that directs high-level expression to myeloid progenitors in transgenic mice. The enhancer is bound and activated by Runx1, Scl, GATA2, C/EBPα, c-Myb, Pu.1, and additional Ets factors in myeloid cells. CRISPR/Cas9-mediated replacement of the wild-type enhancer with a variant mutant in its seven Ets sites leads to 20-fold reduction of *Cebpa* mRNA in the 32Dcl3 myeloid cell line. To determine the effect of deleting the enhancer *in vivo*, we now characterize C57BL/6 mice in which *lox*P sites flank a 688 bp DNA segment containing the enhancer. CMV-Cre mediated germline deletion resulted in diminution of the expected number of viable Enh(f/f);CMV-Cre offspring, with 28-fold reduction in marrow *Cebpa* mRNA but normal levels in liver, lung, adipose, intestine, muscle, and kidney. Cre-transduction of lineage-negative marrow cells *in vitro* reduced *Cebpa* mRNA 12-fold, with impairment of granulocytic maturation, morphologic blast accumulation, and IL-3 dependent myeloid colony replating for >12 generations. Exposure of Enh(f/f);Mx1-Cre mice to pIpC led to 14-fold reduction of *Cebpa* mRNA in GMP or CMP, 30-fold reduction in LSK, and <2-fold reduction in the LSK/SLAM subset. FACS analysis of marrow from these mice revealed 10-fold reduced neutrophils, 3-fold decreased GMP, and 3-fold increased LSK cells. Progenitor cell cycle progression was mildly impaired. Granulocyte and B lymphoid colony forming units were reduced while monocytic and erythroid colonies were increased, with reduced *Pu*.*1* and *Gfi1* and increased *Egr1* and *Klf4* in GMP. Finally, competitive transplantation indicated preservation of functional long-term hematopoietic stem cells upon enhancer deletion and confirmed marrow-intrinsic impairment of granulopoiesis and B cell generation with LSK and monocyte lineage expansion. These findings demonstrate a critical role for the +37 kb *Cebpa* enhancer for hematopoietic-specific *Cebpa* expression, with enhancer deletion leading to impaired myelopoiesis and potentially preleukemic progenitor expansion.

## Introduction

CCAAT/enhancer binding protein α (C/EBPα) is a basic region-leucine zipper transcription factor expressed preferentially within granulocytic and monocytic myeloid cells during hematopoiesis [1]. C/EBPα levels increase as long-term hematopoietic stem cells (LT-HSC) progress to the common myeloid progenitor (CMP) and subsequently to the granulocyte-monocyte progenitor (GMP), with *Cebpa* open reading frame (ORF) deletion preventing GMP formation associated with accumulation of upstream CMP and the Lin^-^Sca-1^+^c-kit^+^ (LSK) stem/progenitor subsets [2, 3]. As GMP mature, high-level C/EBPα expression is required for granulopoiesis while reduced levels allow monopoiesis [4].

C/EBPα expression or activity is commonly diminished in acute myeloid leukemia (AML) cases, including *CEBPA* point mutations impacting trans-activation or DNA-binding, RUNX1-ETO expression reducing *CEBPA* transcription, and C/EBPα(S21) phosphorylation also impairing trans-activation [5].

The *Cebpa* promoter is directly activated by C/EBPα and RUNX1 [6, 7]. In addition, we identified a 440 bp DNA segment centered at +37.5 kb in the murine *Cebpa* gene, with 85% homology to the +42 kb region of the human *CEBPA* locus, harboring enhancer specific H3K4me1 histone marks and together with the promoter capable of directing high-level hCD4 transgene expression to GMP, CMP, and LSK cells but not to multiple non-hematopoietic tissues [7, 8]. Runx1, C/EBPα, Pu.1, Erg, Fli-1, GATA2, Scl, Meis1, and Gfi-1b bind chromatin in the region of this enhancer in hematopoietic cells as determined by ChIP-Seq [9, 10], Runx1, C/EBPα, Pu.1, Fli-1, Erg, Ets1, c-Myb, GATA2, and Scl bind conserved enhancer *cis* elements in gel shift assays, and mutation of the Runx1, C/EBP, Ets, Myb, GATA, or E-box sites each reduce enhancer activity in 32Dcl3 myeloid cells in reporter assays [7, 11]. Mutation of its seven Ets sites led to the greatest reduction in enhancer activity, and CRISPR/Cas9-mediated replacement of the endogenous enhancer alleles with a variant harboring point mutations in these Ets sites led to 20-fold reduced *Cebpa* mRNA expression in 32Dcl3 myeloid cells [11].

To determine whether the +37 kb *Cebpa* enhancer is also critical for regulating *Cebpa* expression *in vivo*, we have now generated and characterized mice in which *lox*P sites flank the enhancer, designated as Enh(f/f) mice. Germline deletion using CMV-Cre revealed marked reduction of *Cebpa* expression in marrow but not in other tissues, including liver, adipose, and lung, that normally express C/EBPα. As germline deletion or use of Vav-Cre to induce hematopoietic-specific deletion led to significant early post-natal lethality, we focused on analysis of adult Enh(f/f);Mx1-Cre mice subjected to pIpC injections to induce enhancer deletion, followed by recovery for four weeks to reestablish homeostasis and to avoid transient pIpC effects. In this model, *Cebpa* mRNA was reduced 14-fold in GMP or CMP and 30-fold in the LSK marrow population associated with a 3-fold reduction in GMP, LSK expansion, LSK/SLAM cell depletion, and impaired granulopoiesis relative to monopoiesis. Erythroid progenitor and platelet expansion and reduced numbers of B lymphoid colony forming units was also observed, with preservation of functional LT-HSC. These findings demonstrate that the +37 kb *Cebpa* enhancer is central to regulation of *Cebpa* transcription and granulopoiesis *in vivo*.

## Methods

### Ethics Statement

This study was carried out in strict accordance with the recommendations in the Guide for the Care and Use of Laboratory Animals of the National Institutes of Health. The protocol (M013M116) was approved by the Johns Hopkins University Animal Care and Use Committee. All efforts were made to minimize suffering.

### Generation of Enhancer-Floxed Mice

The C57BL/6 (B6)-derived 123 kb BAC RP23-375B6 was obtained from CHORI. Recombineering methodology [12] was then utilized to transfer a 6,950 bp segment containing the 439 bp enhancer, a 940 bp 3’ homology arm, and a 5,520 bp 5’ homology arm to pBluescript II, followed by insertion of a *lox*P site 214 bp upstream and a *frt*-PGK-Neo-*frt*-*lox*P cassette derived from plasmid PL451 35 bp downstream of the enhancer, thus floxing a 688 bp genomic DNA segment. After removal of vector sequences, the plasmid insert was provided to the Johns Hopkins Transgenic Core facility, which generated multiple G418-resistant B6 BL-1 embryonic stem cell (ESC) lines after electroporation. These were screened by 3’PCR using a forward primer near the 3’ end of the Neo cassette and a reverse primer distal to the 3’ homology arm. Homologous recombination was confirmed by Southern blotting after *Spe*I digestion of genomic DNA, as described [13]. The 5’ probe was a 1.3 kb *Kpn*I/*Hind*III fragment centered 4.1 kb 5’ to the enhancer, and the 3’ probe was a 0.9 kb *Bam*HI/*Xho*I fragment encompassing the 3’ homology arm. Targeted B6 ESC lines were then utilized to generate chimeric mice after injection into B6-albino blastocysts. Briefly, ~12–15 targeted ESC were injected into blastocyst stage embryos obtained from superovulated B6(Cg)-*Tyrc-2J*/J females (Jackson Laboratories, #58). Following injection, surviving embryos were surgically transferred to oviducts of psuedopregnant ICR females (~15 embryos/female). Chimeric offspring were then bred to B6-albino mice and offspring with fully black fur were screened by tail clip DNA PCR for presence of the knockin (KI) DNA using primers that flank the 5’ *lox*P site:

loxP5-F: 5’-ACCTTCCGTGCTCAAGTCTG and

loxP5-R: 5’-AAGTCCCCTTTGCCAGACAC, followed by 1.5% agarose gel electrophoresis.

Successfully targeted mice were then bred to homozygosity. Mx1-Cre (#3556), female CMV-Cre (#6054), female ROSA26-FLPo (#12930), and Vav-Cre (#8610) mice were obtained from Jackson Laboratories. Cre DNA was detected using:

Cre-F: 3’-GTCCAATTTACTGACCGTACAC and

Cre-R: 5’-CTGTGACTTGGTCGTGGCAGC.

Deletion of both floxed alleles by CMV-Cre was determined by absence of the 5’ *lox*P site and by detection of a band of appropriate size using primers upstream of the 5’ *lox*P site and downstream of the 3’ *lox*P site:

EnhΔ-F: 5’-CCCAAGACAGCCAGGTTAGGAGTTCC and

EnhΔ-R: 5’-ACATGATGTCCCGGAGAACAGAGCC.

Bialleleic deletion of the Neo cassette after FLPo expression was assessed using primers:

Frt5-F: 5’-GGTCTGAAGAGGAGTTTACGTCC located just downstream of the 5’ *frt* site and

PGK-R: 5’-AGAGGAGAACAGCGCGGCAG located in the PGK promoter and primers:

Enh-F: 5’-CCACATCACACGGGGCCTGC and

3Arm-R: 5’-ACACCAAGAGCTAAGAGGACACCCC flanking the entire *frt*-PGK-Neo-*frt* cassette. 8–12 wk old Enh(f/f);Mx1-Cre mice were injected intraperitoneally with 500 μg of pIpC (Sigma) every other day for 6 doses. Blood or marrow was isolated 4 wks later for analysis.

### Retroviral Transduction and Progenitor Assays

293T cells were cultured in Dulbecco’s modified Eagle medium (DMEM) with 10% heat-inactivated fetal bovine serum (HI-FBS). For *in vitro* studies, marrow isolated from Enh(f/f) or wild-type (WT) mice injected intraperitoneally with 150 mg/kg 5-fluorouracil (5-FU) 6 days earlier was subjected to red cell lysis with NH_4_Cl and cultured for 1 day in 10 ng/mL murine IL-3, 10 ng/mL murine IL-6, and 10 ng/mL murine SCF (Peprotech) followed by addition of 4 μg/mL Polybrene and retroviral supernatants obtained from 293T cells transduced with 12 μg of pBabePuro or pBabePuro-Cre, 3 μg of pkat2ecopac, and 35 μL Lipofectamine 2000 (Invitrogen) per 100 mm dish as described [7]. Three days later, 2 μg/mL puromycin was added, and after 2 additional days viable cells isolated with Lympholyte M (Cedarlane Labs) were either plated in methylcellulose or subjected to lineage-depletion using biotin-conjugated B220, Gr-1, CD11b, Ter119, and CD3 mouse Lineage Cocktail (BD Pharmingen), anti-biotin microbeads, and MACS columns (Miltenyi Biotec) and placed in liquid culture with IMDM, 10% HI-FBS and IL-3, IL-6, and SCF. Myeloid colonies were enumerated 7–8 days later based on colony morphology. Myeloid colonies were also obtained using 30 ng/mL human G-CSF (Amgen), 30 ng/mL murine M-CSF, or 30 ng/mL murine GM-CSF (Peprotech). BFU-E were enumerated on day 10 after culture in Methocult M3120 (1% final concentration) with IMDM, 2 mM glutamine, 55 nM β-mercaptoethanol, 10% plasma-derived serum (Animal Technologies), 20% BIT (Stem Cell Technologies), 5% PFHM-II (Invitrogen), and 10 U/mL (100 ng/mL) murine erythropoietin (EPO). B lymphoid CFU were enumerated 7 days after culture in Methocult 3630, which contains IMDM, HI-FBS, and human IL-7. For myeloid colony replating, CFUs were pooled, washed with PBS, and replated at 1E3 cells/mL every 7 days. Cell morphology was assessed by Wright-Giemsa staining of cytospun cells. Photomicrographs were taken using a Zeiss Axiophot microscope (Carl Zeiss), a Kontron Electronik Progress 3012 camera (Kontron), and a 63X/1.40 NA oil objective.

### Quantitative RNA Analysis and Western Blotting

RNA from hematopoietic cells was prepared using NucleoSpin RNA II, with use of RNase-free DNase (Machery-Nagel). Tissues were homogenized in Trizol using Tissue-Tearor (United Laboratory Plastics); RNA was extracted using chloroform, isopropanol precipitated, and further purified using NucleoSpin RNA II. First strand cDNA was prepared using AMV reverse transcriptase (Promega) and oligodT primer at 42°C for 1 hr. Quantitative PCR was carried out using 5–25 ng of each cDNA using iQ SYBR Green supermix (Bio-Rad). *Cebpa*, *Cebpg*, *GMCSFRa*, and ribosomal subunit *mS16* internal control primers were:

Cebpa-F: 5’-TGGATAAGAACAGCAACGAG,

Cebpa-R: 5’-TCACTGGTCAACTCCAGCAC,

Cebpg-F: 5’-GCGCAGAGAGCGGAACAA,

Cebpg-R: 5’-GTATCTTGAGCTTTCTGCTTGCT,

GMCSFRa-F: 5’-CCAGGGATCAGGGACAAGG,

GMCSFRa-R: 5’-CCTGTCAGTCACGTTGGGG,

mS16-F: 5’-CTTGGAGGCTTCATCCACAT, and

mS16-R: 5’-ATATTCGGGTCCGTGTGAAG.

Additional primer pairs were as described [4, 8]. Western blotting for C/EBPα and β-actin was carried out as described [7].

### FACS Analysis and Flow Cytometry

Antibodies were from Pharmingen unless otherwise specified. Peripheral blood was obtained by lancing the facial vein and collecting several drops into EDTA microtainers (Pharmingen). Blood counts were obtained using a Hemavet 50FS. Blood elements were enumerated using PE or PerCP-Cy5.5-anti-CD3 (145-2C11), PE-anti-B220 (RA3-6B2), APC-anti-CD19 (1D3), APC, PE or PerCP-Cy5.5-anti-CD11b (M1/70), PE or PerCP-Cy5.5-anti-Ter119 (Ter119), and PerCP-Cy5.5 or APC-anti-Gr-1 (RB6-8C5, Biolegend). Stem and progenitor cells were enumerated using biotin-anti-Lineage Cocktail, PerCP-Cy5.5-streptavidin, APC-anti-c-Kit (2B8) and PE-Cy7-anti-Sca-1 (D7, eBioscience), in addition to PE-anti-CD16/CD32 (FcγR, 2.4G2) and Brilliant Violet 421-anti-CD34 (RAM34) for CMP, GMP, and MEP, PE-Texas Red-anti-CD127 (IL7R, SB199) for CLP, or Brilliant Violet 421-anti-CD34 and PE-anti-CD135 (FLT3, A2F10.1) for MPP, ST-HSC, and LT-HSC. Alternatively, LSK cells were stained using PE—anti-CD150 (Q38-480) and Brilliant Violet 421–anti-CD48 (HM48-1) for LSK/SLAM LT-HSC. Marrow subsets for RNA analysis were obtained after lineage-depletion and antibody staining via a FACSAria II cell sorter (BD Biosciences).

### Cell Proliferation and Apoptosis Assays

For cell cycle analysis using the BrdU Flow kit (BD Pharrmingen), mice were given a single intraperitoneal injection of 5-bromodeoxyuridine (BrdU; 100 μg/g). 3 hrs later, bone marrow was isolated from femurs, tibias, iliac crest and spine. After red cell lysis, the cells were lineage depleted as above. Lineage negative cells were stained with LIVE/DEAD Fixable Aqua (Life Technologies) and for LSK, CMP, GMP, and MEP as above. The fixation, DNase treatment, and staining with FITC anti-BrdU and 7AAD were per the BrdU Flow kit protocol. For quiescence analysis, lineage depleted marrow was stained with LIVE/DEAD Fixable Aqua and surface makers for LSK, CMP, GMP, and MEP, fixed, treated with DNase, and stained with FITC-anti-Ki67 (eBioscience, SolA15) and 7AAD. For analysis of apoptosis and cell death, marrow without red cell lysis was lineage depleted using biotin-conjugated mouse Lineage Cocktail (BD Pharmingen), MojoSort Streptavidin Nanobeads (Biolegend), and EasySep Magnet (Stemcell Technology), followed by staining for progenitor subsets and with Alexa Fluor 488-anti-Annexin V (Life Technologies) and 7AAD.

### Transplantation Studies

Enh(f/f);Mx1-Cre CD45.2^+^ marrow cells, obtained 4 wks after pIpC exposure, were transplanted by tail vein injection at a 1:1 ratio with 2E5 CD45.1^+^ WT competitor cells into syngeneic WT CD45.1^+^ recipient mice irradiated to 950 cGy. Mice were euthanized at 19 wks, and marrow and peripheral blood cells were analyzed using PE- or FITC-anti-CD45.1 (A20), and APC-anti-CD45.2 (104, Biolegend) and additional antibodies as above. 1E6 marrow cells from primary recipients were transplanted into irradiated secondary recipients, one secondary mouse per primary mouse, followed by similar blood analysis 16 wks later.

### Statistics

Means and standard deviations (SD) are shown. The Student *t* test was used for statistical comparisons.

## Results

### Generation of Mice with Floxed +37 kb *Cebpa* Enhancer Alleles

**Fig 1:**
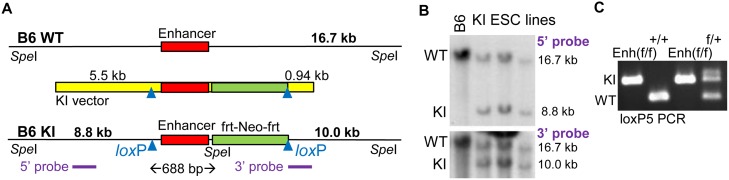
Generation of B6 Mice with Floxed *Cebpa* +37 kb Enhancer Alleles. **A**) Diagram of a wild-type genomic allele in the vicinity of the +37 kb *Cebpa* enhancer (B6 WT), the knockin (KI) vector, and a targeted genomic allele (B6 KI). The conserved 439 bp enhancer is flanked by a *frt*-PGK-Neo-*frt* cassette and two *lox*P sites. Cre-mediated deletion of the DNA between the *lox*P sites will remove the enhancer, 214 bp of DNA 5’ to the enhancer, and 35 bp of DNA 3’ to the enhancer, 688 bp in total. Positions of *Spe*I sites, fragment sizes expected after *Spe*I digestion, and locations of the 5’ and 3’ probes used for Southern blotting are also shown. **B**) Southern blots obtained after *Spe*I digestion of genomic DNA isolated from parental B6 BL-1 ESC (B6) or from three targeted lines (KI ESC lines) using the 5’ or 3’ probes. Locations and sizes of fragments derived from the WT and KI alleles are indicated. **C**) PCR of tail snip DNA, using loxP5 primers spanning the 5’ *lox*P site, from homozygous Enh(f/f), WT (+/+), and heterozygous (f/+) mice obtained after breeding. Locations of bands obtained from the KI and WT alleles are indicated.

A targeting construct for homologous knockin (KI vector) was assembled in which the 439 bp +37 kb *Cebpa* enhancer, 214 bp of genomic DNA upstream of the conserved enhancer segment, 35 bp of downstream DNA, and a downstream *frt*-PGK-Neo-*frt* cassette are flanked by *lox*P sites (Fig 1A). This construct also includes a 5.5 kb 5’ homology arm and a 940 bp 3’ homology arm. The 3’ homology arm length was limited by a microsatellite repeat. The genomic DNA segments were derived from a B6 BAC. Of note, 129 strain BAC clones spanning the enhancer were not available. We positioned the 5’ *lox*P site 214 bp upstream of the enhancer, rather than closer, due to a B6:129 homology gap at this location, to potentially facilitate 129 ESC cell targeting. The 3’ arm was identical between B6 and 129 DNA, but the 5’ arm contains 10 single nucleotide differences and a 4 bp gap`.

KI vector electroporation into 129 ESC followed by G418 selection yielded only one successful homologous replacement (HR) event among 376 subclones screened by 3’ PCR, whereas electroporation into a B6 ESC line yielded six subclones with HR among 176 lines screened. Genomic DNA isolated from the parental B6 ESC line and from three targeted B6 lines was digested with *Spe*I and subjected to Southern blotting using a 1.3 kb probe located in the 5’ homology arm or a 0.9 kb probe encompassing the 3’ homology arm (Fig 1B). A 16.7 kb band was detected with either probe in all four lines, representing unmodified genomic DNA. In addition, 8.8 kb or 10.0 kb bands were detected in the three KI ESC lines with the 5’ or 3’ probe, respectively, indicating presence of a properly targeted allele. Two of these lines were microinjected into albino blastocysts, yielding chimeric offspring with black and white coat colors. These were bred to albino mice, and tail snip DNAs from offspring with all black coat color were screened by PCR with a primer pair surrounding the 5’ *lox*P site. Heterozygous Enh(f/+) mice were then bred to generate homozygous Enh(f/f) mice, as assessed also by genomic DNA PCR using the loxP5 primer pair (Fig 1C).

### Effect of *In Vitro* Enhancer Deletion on *Cebpa* RNA Expression and Myelopoiesis

**Fig 2:**
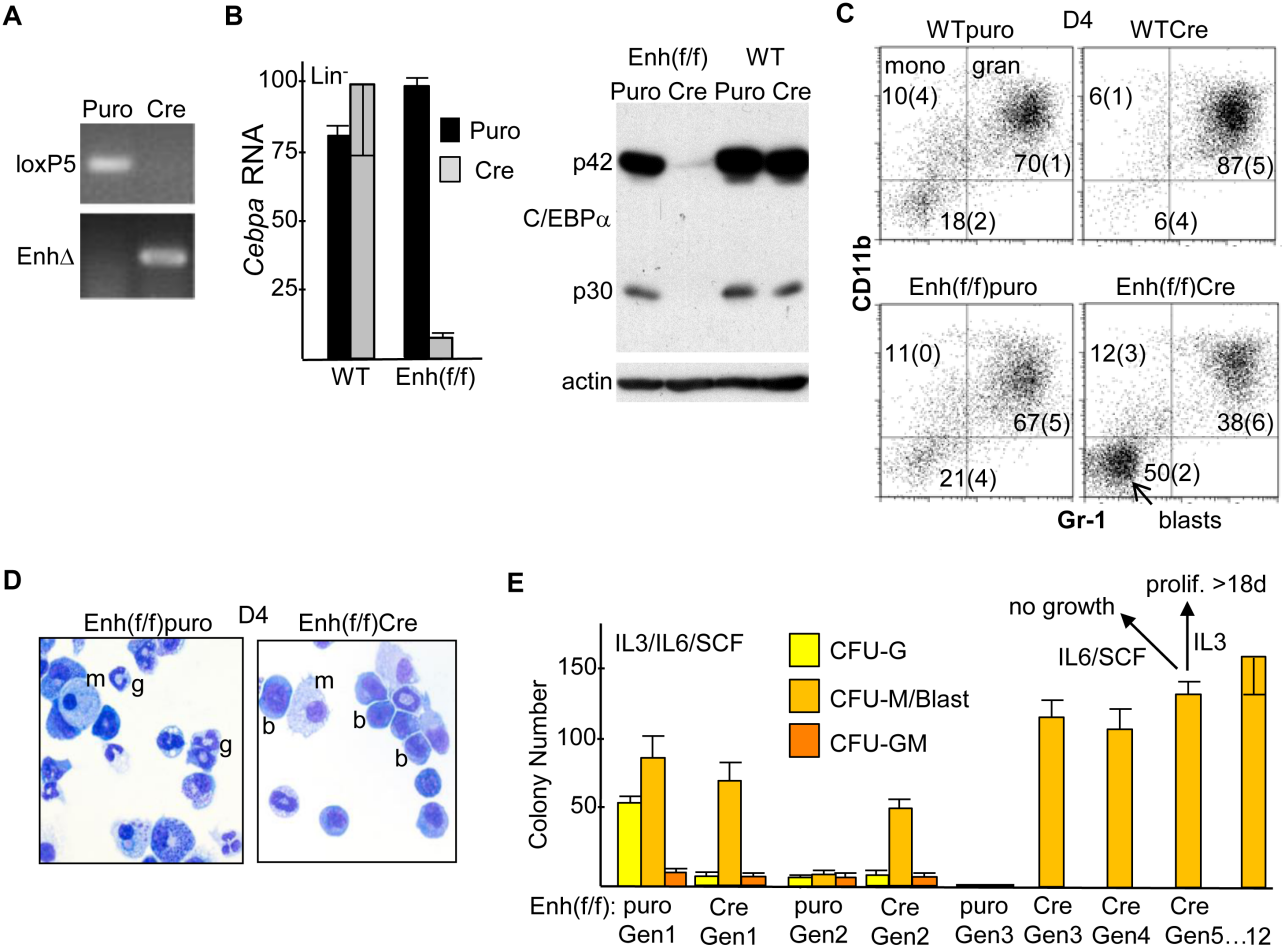
Effect of *in vitro* Enhancer Deletion on *Cebpa* Expression and Myelopoiesis. **A**) Mononuclear marrow cells from WT or Enh(f/f) mice were placed in IMDM/FBS with IL-3, IL-6 and SCF for 24 hr, transduced with pBabePuro (Puro) or pBabePuro-Cre (Cre) for 48 hr, puromycin selected for an additional 48 hr, and finally lineage-depleted. Genomic DNA was then subjected to PCR using the loxP5 or EnhΔ primer pairs followed by agarose gel electrophoresis and visualization by ethidium bromide staining. **B**) Total cellular RNAs were analyzed for *Cebpa* and large ribosomal subunit *mS16* mRNA expression. *Cebpa* RNA expression, normalized using *mS16* expression and set to 100 for WT marrow transduced with Cre, is shown (left, mean and SD from 3 determinations). Total cellular proteins isolated from the same groups of Lin^-^ cells were subjected to Western blotting for C/EBPα and β-actin; locations of the p42 and p30 C/EBPα alternative translation variants are indicated (right). **C**) Lin^-^ cells were placed in liquid culture with IMDM/FBS, IL-3, IL-6, and SCF and analyzed for surface CD11b and Gr-1 expression on day 4 (D4; mean and SD from three determinations). **D**) The morphology of Puro- or Cre-transduced Enh(f/f) cells from these cultures was assessed on D4 by Wright’s Giemsa staining; g—granulocyte; m—monocyte; b—blast. **E**) Lin^-^ cells were cultured similarly in methylcellulose at 1E3 cell/mL, and myeloid CFUs were enumerated 7–8 days later (Gen1). CFU cells were then collected, washed with PBS, replated at 1E3 cells/mL, and analyzed similarly each 7 days (Gen 2 to Gen 12). In addition, a proportion of Gen5 cells were evaluated for their ability to proliferate in liquid culture in IMDM/FBS with IL-6/SCF or IL-3.

Mononuclear marrow cells from 8–12 wk old WT or Enh(f/f) mice exposed six days earlier to 5-FU were cultured in IMDM/FBS with the myeloid cytokines IL-3, IL-6, and SCF, transduced with pBabePuro (Puro) or pBabePuro-Cre (Cre), subjected to puromycin selection followed by removal of dead cells, and finally lineage-depleted. PCR analysis of DNA from Cre-transduced cells demonstrates highly efficient enhancer deletion, as indicated by complete loss of the floxed, KI 5’ *lox*P site PCR product using the loxP5 primer pair and gain of a PCR product resulting from enhancer deletion (EnhΔ, Fig 2A). Quantitative RT-PCR analysis demonstrated equivalent *Cebpa* mRNA expression in Puro-transduced WT versus Enh(f/f) cells, indicating lack of effect of the PGK-Neo cassette on *Cebpa* expression, equivalent expression in Puro- compared with Cre-transduced WT cells, indicating lack of effect of Cre on *Cebpa* expression, and 12-fold average reduction in *Cebpa* RNA in Enh(f/f) cells transduced with Cre versus Puro, indicating a key role for the +37 kb enhancer in regulating myeloid cell autonomous *Cebpa* expression *in vitro* (Fig 2B, left). The same Puro- or Cre-transduced, puromycin-selected, lineage-depleted samples were also subjected to Western blotting for C/EBPα and β-actin (Fig 2B, right). Cre-transduction of Enh(f/f) marrow led to marked reduction in C/EBPα protein, both its full-length p42 and shorter p30 isoforms.

Transduced, puromycin-selected lineage-negative (Lin^-^) cells were also placed in liquid culture with IL-3, IL-6, and SCF. Four days later (D4), the cells were analyzed using FACS for CD11b and Gr-1 expression (Fig 2C). Under these culture conditions, CD11b^+^Gr-1^-^ cells represent monocytes and CD11b^+^Gr-1^+^ cells represent granulocytes, as we previously confirmed using additional FACS antibodies [7]. Cre-transduction of Enh(f/f) cells led to ~2-fold reduction in the proportion of granulocytes and a 2.5-fold increase in an immature CD11b^-^Gr-1^-^ “blast” population, compared with Puro-transduced Enh(f/f) cells. Morphologic evaluation of these populations confirmed reduction of mature granulocytes and an increase in immature blastic cells in response to enhancer deletion (Fig 2D). Finally, transduced Lin^-^ cells were placed in methylcellulose culture with IL-3, IL-6, and SCF. Enumeration of first generation (Gen1) colony-forming units (CFU) demonstrated marked reduction in CFU-G in response to enhancer deletion, with little effect on CFU-M or CFU-GM (Fig 2E). The ability of these colonies to replate for successive generations in the same cytokines was then evaluated. Puro-transduced myeloid CFU did not replate past Gen2. In striking contrast, Cre-transduced CFU replated for at least 12 generations, with the morphology of the large majority of CFU cells past the 5^th^ generation having a blastic appearance. In addition to replating in methylcellulose, 5^th^ generation CFU cells were placed in liquid culture with IMDM/FBS and either IL-6/SCF or IL-3. The cells did not proliferate in IL-6/SCF and rapidly died, whereas the cells proliferated continuously for at least 18 days in IL-3, increasing ~2-fold each day. Together, these data indicate that reduced *Cebpa* expression consequent to +37 kb enhancer deletion impairs hematopoietic cell autonomous granulopoiesis *in vitro*, leading to preservation of immature myeloid progenitors capable of long-term, IL-3-dependent proliferation without complete terminal maturation, a preleukemic phenotype.

### Effect of *In Vivo* Enhancer Deletion on *Cebpa* mRNA Expression

**Fig 3:**
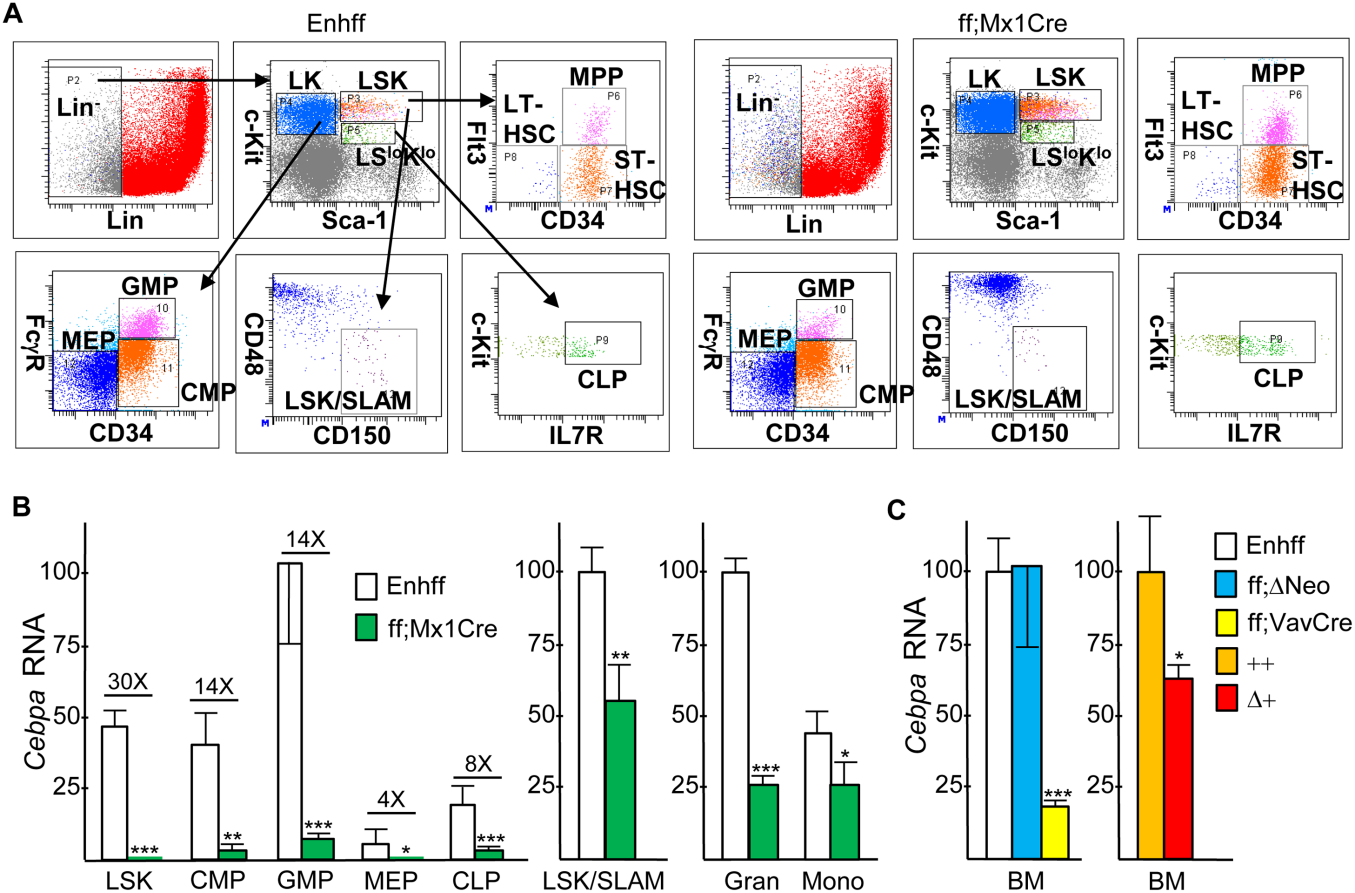
Effect of *in vivo* Enhancer Deletion on *Cebpa* Hematopoietic Expression. **A**) Representative FACS analyses of marrow stem/progenitor subsets. GMP, CMP, and MEP were analyzed within the Lin^-^Sca-1^-^c-kit^+^ (LK) subset, CLP were analyzed within the Lin^-^Sca-1^lo^c-kit^lo^ (LS^lo^K^lo^) subset, and MPP, ST-HSC, LT-HSC, or LSK/SLAM cells were enumerated within the Lin^-^Sca-1^+^c-kit^+^ (LSK) subset. **B**) Total cellular RNAs from the LSK, CMP, GMP, MEP, CLP, LSK/SLAM, granulocyte, or monocyte marrow subsets isolated from Enh(f/f) or Enh(f/f);Mx1-Cre mice that had been subjected to pIpC injections and allowed to recover for 4 wks were analyzed for relative *Cebpa* mRNA expression, normalized to *mS16* mRNA expression (mean and SD from three determinations). **C**) Relative *Cebpa* mRNA expression was analyzed similarly from marrow mononuclear cells isolated from Enh(f/f) mice versus mice lacking both PGK-Neo cassettes (ff;ΔNeo) or Enh(f/f);Vav-Cre mice and from wild-type (++) versus Enh(f+);CMV-Cre (Δ+) mice (n = 3).

To evaluate the *in vivo* effect of *Cebpa* +37 kb enhancer deletion on *Cebpa* expression in adult hematopoietic cells, we followed the example of investigators studying the *in vivo* consequences of floxed *Cebpa* ORF deletion [3, 14, 15] and generated Enh(f/f);Mx1-Cre mice. The activity of the interferon-responsive Mx1 promoter can be induced by double-stranded pIpC RNA injections, leading to efficient Cre induction in marrow cells and variable induction in other tissues. Enh(f/f);Mx1-Cre mice or Enh(f/f) littermates received six pIpC injections over a 12 day period. Four weeks after the last pIpC injection, to ensure maximal deletion efficiency, complete recovery from acute effects of pIpC, and reestablishment of hematopoietic homeostasis, mononuclear marrow cells were subjected to flow cytometry to allow isolation of the LSK, CMP, GMP, MEP, and LSK/SLAM populations. Representative FACS analyses of these and additional marrow hematopoietic stem/progenitor subsets, obtained from an Enh(f/f) and an Enh(f/f);Mx1-Cre mouse, is shown (Fig 3A). After total cellular RNA isolation, *Cebpa* expression was evaluated via RT-PCR (Fig 3B). Due to their limited cell numbers, LSK/SLAM cells were obtained from a separate set of mice; in addition, marrow granulocytes and monocytes were isolated from a third set of mice. As expected, in marrow subsets isolated from Enh(f/f) mice, *Cebpa* mRNA increased as LSK or CMP progressed to GMP and was minimal in MEP. Reduced but evident *Cebpa* in CLP may in part represent expression in a B/myeloid CLP subset. *Cebpa* mRNA was reduced upon Cre-mediated enhancer deletion by 30-fold, on average, in the LSK population, 14-fold in CMP or GMP, 4-fold in MEP, 8-fold in CLP, 1.5-fold in LSK/SLAM cells, 4.6-fold in granulocytes, and 1.6-fold in monocytes. These data indicate a critical dependence upon the presence of the +37 kb *Cebpa* enhancer for *Cebpa* mRNA expression in LSK, CMP, or GMP, intermediate dependence in CLP, and only mild dependence in the LSK/SLAM subset. Greater reduction of *Cebpa* RNA in GMP compared to granulocytes or monocytes may reflect maturation from a small number of GMP lacking complete enhancer deletion.

Using ROSA26-FLPo mice, we generated mice lacking the PGK-Neo cassette but retaining the *lox*P sites flanking the enhancer. *Cebpa* RNA expression in marrow mononuclear cells from these mice was equivalent to that from Enh(f/f) mice retaining the Neo cassette (Fig 3C). These data are consistent with the finding that Puro-transduced WT and Enh(f/f) Lin^-^ marrow cells express equivalent levels of *Cebpa* mRNA.

We also generated Enh(f/+);Vav-Cre mice and mated these together in an effort to obtain Enh(f/f);Vav-Cre offspring. The Vav promoter is expressed throughout hematopoiesis, beginning during the fetal liver stage of development, but not in non-hematopoietic tissues. However, Enh(f/f);Vav-Cre offspring were obtained at 25% of the expected Mendelian ratio, and the three such mice identified were runted, potentially reflecting increased susceptibility of neonates to the infectious consequences of marked neutropenia. Analysis of *Cebpa* mRNA expression in marrow cells from these Enh(f/f);Vav-Cre mice demonstrated 6-fold reduced expression (Fig 3C), further confirming that the +37 kb *Cebpa* enhancer acts in a hematopoietic autonomous manner to play a key role in regulating *Cebpa* transcription.

**Fig 4:**
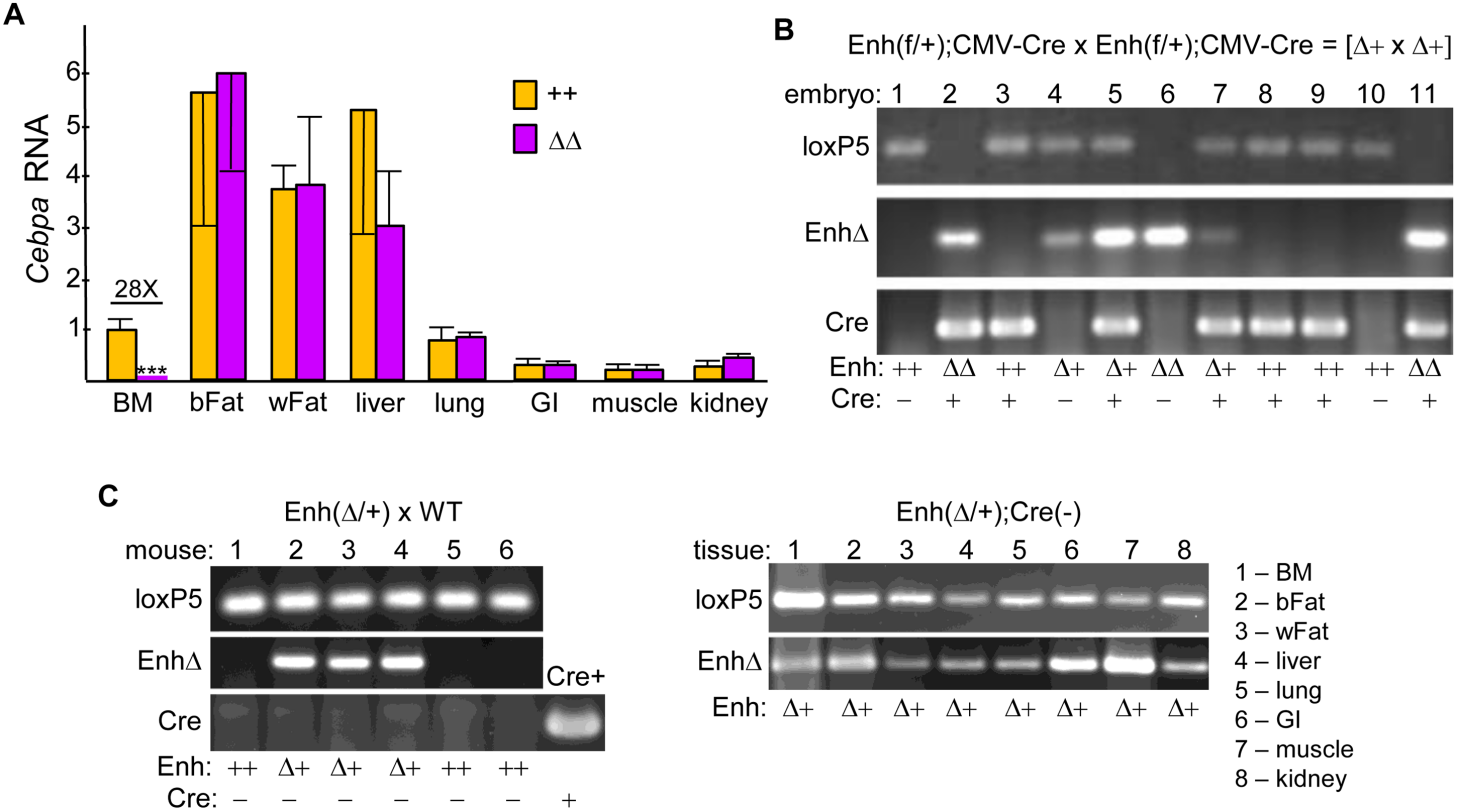
Effect of Germline Enhancer Deletion on *Cebpa* Expression in Non-Hematopoietic Tissues. **A**) Total RNAs from marrow mononuclear cells, brown fat (bFat), white fat (wFat), liver, lung, small intestine (GI), skeletal muscle, or kidney from wild-type (++) and Enh(f/f);CMV-Cre (ΔΔ) mice were analyzed similarly for relative *Cebpa* expression (n = 3). **B**) Total embryo DNA from a litter obtained at E16.5 from a cross between Enh(f/+);CMV-Cre (Δ+) mice was subjected to PCR using the loxP5, EnhΔ, and Cre primer pairs, followed by agarose gel electrophoresis and ethidium bromide staining. Enhancer and Cre genotypes are indicated. **C**) Tail DNA from a litter of 4 wk pups obtained from a cross between a WT and Δ+ mouse, both lacking CMV-Cre, was analyzed similarly (left). DNA from a mouse harboring Mx1-Cre served as a positive control for the Cre PCR (Cre+). DNAs from adult tissues of one of the Δ+ pups were also analyzed using loxP5 and EnhΔ5 PCR (right). * - p<0.05, ** - p<0.01, *** - p<0.001.

Finally, to assess the effect of enhancer deletion in non-hematopoietic tissues, we utilized CMV-Cre, which provides germline deletion [16]. In addition to marrow myeloid cells, *Cebpa* is expressed prominently in adipocytes, hepatocytes, and type II pneumocytes [17, 18]. As with Vav-Cre, upon mating Enh(f/+);CMV-Cre, designated Δ+, mice few offspring with homozygous enhancer deletion (ΔΔ) were obtained, 40% of the predicted number, and those obtained were again smaller than their littermates. *Cebpa* marrow RNA was reduced 1.5-fold, on average, in Δ+ vs Enh(f/f) control (++) littermates (Fig 3C). RNAs isolated from marrow or from seven non-hematopoietic tissues from Enh(f/f);CMV-Cre (ΔΔ) or age-matched wild-type (++) mice were subjected to analysis of *Cebpa* expression (Fig 4A). *Cebpa* mRNA was reduced 28-fold in total marrow mononuclear cells, on average. In contrast, no significant reduction was seen in brown fat, white fat, liver, lung, small intestine, skeletal muscle, or kidney.

To confirm that CMV-Cre mediates germline enhancer deletion of the floxed *Cebpa* enhancer, we first isolated DNA from a litter of E16.5 embryos derived from a cross between Enh(f/+);CMV-Cre (Δ+) parents, followed by PCR analysis for the 5’ *lox*P site, non-floxed WT product (loxP5), enhancer deletion (EnhΔ), and Cre (Fig 4B). Embryos were used to increase the likelihood of obtaining homozygous enhancer deletion. Presence of the loxP5 and not the EnhΔ band indicates enhancer genotype ++; presence of both bands indicates heterozygous deletion or Δ+, and presence of only the EnhΔ band indicates homozygous deletion or ΔΔ. Embryo 4 lacks Cre but has heterozygous Δ+ enhancer deletion, and embryo 6 lacks Cre but has homozygous ΔΔ enhancer deletion. Presence of enhancer deletion on one or both alleles in the absence of Cre indicates that in a prior generation CMV-Cre mediated germline enhancer deletion, which would then be passed on to subsequent offspring in all tissues. Second, we similarly evaluated tail DNA from 4 wk old offspring obtained from a cross between a wild-type B6 mother (Jackson Laboratories) and an Enh(Δ+) father who similar to Embryo 4 lacked CMV-Cre (Fig 4C, left). Three pups had an Enh(Δ+) genotype, despite absence of Cre in their or their parent’s genomes, indicating continued inheritance of enhancer deletion via the germline. Moreover, further analysis of one of these mice indicated that each of eight tissues analyzed lacked an enhancer allele (Fig 4C, right). Taken together, these RNA and DNA data indicate that the +37 kb *Cebpa* enhancer acts specifically in hematopoietic cells compared to those non-hematopoietic tissues analyzed.

### Effect of *Cebpa* Enhancer Deletion on Hematopoietic Lineage Development

**Fig 5:**
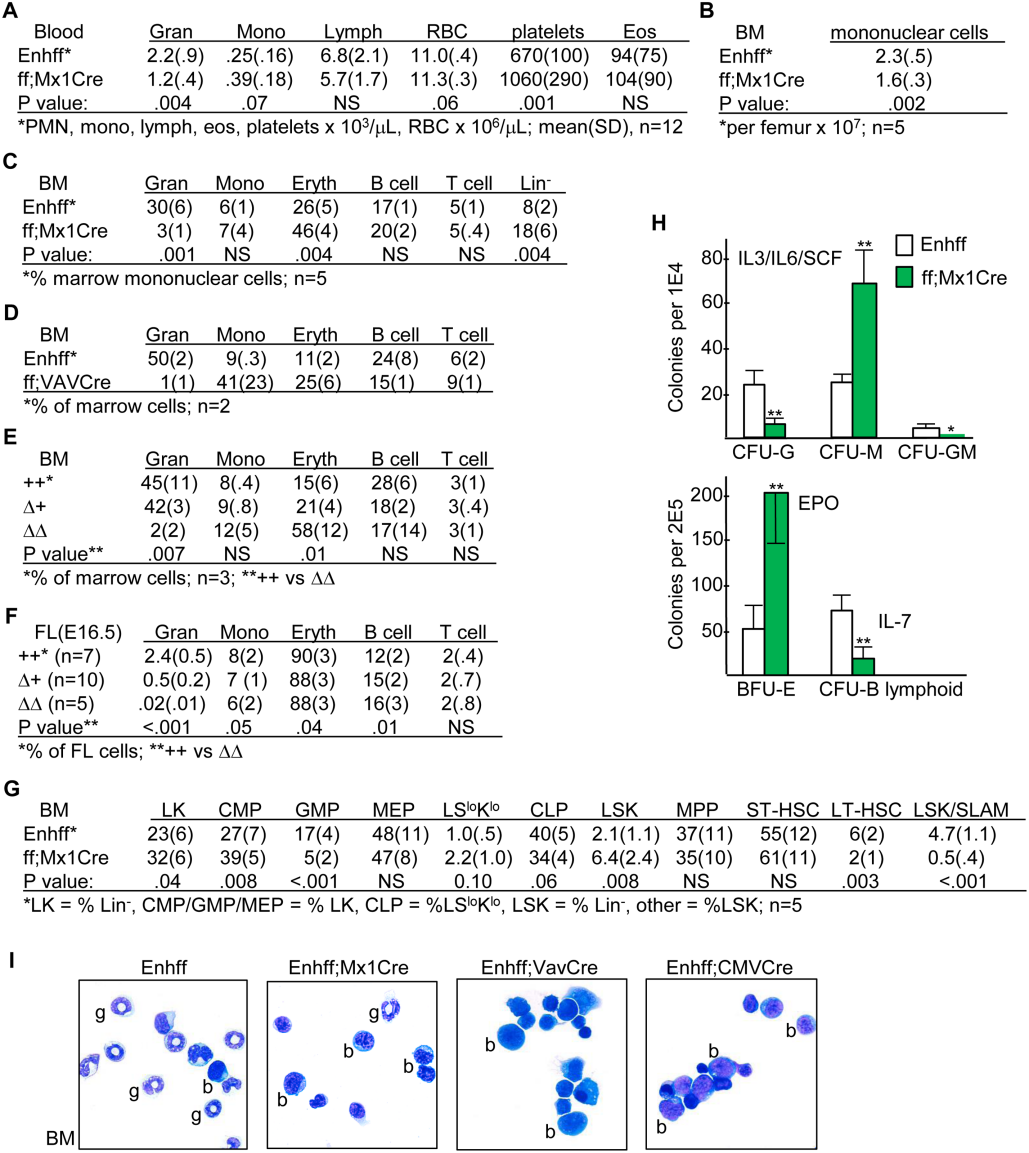
Effect of *in vivo* Enhancer Deletion on Hematopoiesis. **A**) Peripheral blood counts from 14–16 wk old Enh(f/f) and Enh(f/f);Mx1-Cre mice obtained 4 wks after completion of pIpC injections. **B**) Total marrow cellularity from Enh(f/f) versus Enh(f/f);Mx1-Cre mice exposed 4 wks earlier to pIpC. **C**) Marrow CD11b^+^Gr-1^+^ granulocytes, CD11b^+^Gr-1^-^ monocytes, Ter119^+^ erythroid cells, B220^+^ B cells, CD3^+^ T cells, or Lin^-^ cells lacking these markers from these same mice. **D)** Marrow granulocytes, monocytes, erythroid, B and T cells from 4 wk old Enh(f/f) and littermate Enh(f/f);Vav-Cre mice. **E**) Marrow granulocytes, monocytes, erythroid, B and T cells from 5 wk old Enh(f/f) (++), Enh(f/+);CMV-Cre (Δ+), and Enh(f/f);CMV-Cre (ΔΔ) mice. **F**) E16.5 FL cells obtained from embryos of indicated genotypes derived from Enh(f/+);CMV-Cre (Δ+) parents were subjected to FACS analysis for these same hematopoietic subsets. **G**) Marrow stem/progenitor subsets, as determined by FACS analysis, from the same mice evaluated in B and C. **H**) Myeloid, BFU-E, and B lymphoid CFUs were in enumerated after culture at 1E4 cells/mL in methylcellulose in the presence of IL-3/IL-6/SCF or at 2E5 cells/mL in EPO or IL-7, respectively (mean and SD from three determinations). **I**) Morphology of marrow obtained from Enh(f/f) or Enh(f/f);Mx1-Cre mice 4 wks after pIpC injections and from Enh(f/f);Vav-Cre or Enh(f/f);CMV-Cre mice.

Blood counts from Enh(f/f) and Enh(f/f);Mx1-Cre mice obtained 4 wks after pIpC injections were enumerated (Fig 5A). Neutrophils were reduced ~2-fold, platelets were increased 1.5-fold, and monocytes were increased 1.6-fold in the absence of the *Cebpa* +37 kb enhancer, though the monocytosis did not reach statistical significance. Eosinophils were unchanged. Total marrow mononuclear cellularity was reduced 1.4-fold upon enhancer deletion (Fig 5B). Based on FACS analysis, marrow CD11b^+^Gr-1^+^ neutrophils were reduced 10-fold, Ter119^+^ erythroid cells were increased 1.8-fold, and the Lin^-^ subset was increased 2.3-fold (Fig 5C). B220^+^ B cells, 80–85% of which co-expressed CD19, were unchanged, as were CD3^+^ T cells. A similar pattern of neutropenia and erythroid lineage expansion seen in the two Enh(f/f);Vav-Cre and three Enh(f/f);CMV-Cre mice available for analysis (Fig 5D and 5E). In addition we analyzed the fetal livers (FL) of 22 E16.5 embryos, seeing 5-fold reduced granulocytes in Δ+ and virtual absence of granulocytes in ΔΔ embryos (Fig 5F). In the FL, erythropoiesis is dominant, representing ~90% of hematopoietic cells, and there was little difference in the percentage of erythroid cells between ++ and ΔΔ embryos. Of note, the ratio of ++:Δ+:ΔΔ embryos, 7:10:5, was near Mendelian, consistent with prior evaluation of *Cebpa* ORF +/+ vs +/- vs -/- newborns [19] and with the idea that neonatal lethality due to sepsis accounts for the markedly reduced numbers of Enh(f/f);CMV-Cre (ΔΔ) or Enh(f/f);Vav-Cre mice obtained upon weaning.

Regarding marrow stem/progenitor subsets, Mx1-Cre mediated enhancer deletion increased CMP 1.4-fold, reduced GMP 3.4-fold, increased LSK 3-fold, reduced LSK;CD34^-^Flt3^-^ LT-HSC 3-fold, and reduced the LSK/SLAM population 9.4-fold, on average (Fig 5G). In methylcellulose culture with IL-3/IL-6/SCF, enhancer deletion reduced CFU-G 5-fold while increasing CFU-M 3-fold; BFU-E obtained by culture with EPO were increased 4-fold, and B lymphoid CFU obtained in IL-7 were reduced 4-fold (Fig 5H). Morphologic analysis of marrow from Enh(f/f) compared with Enh(f/f);Mx1-Cre, Enh(f/f);Vav-Cre, or Enh(f/f);CMV-Cre mice demonstrates marked reduction in neutrophils and increased cells with blast morphology in the absence of the *Cebpa* enhancer, the latter consistent with the expanded Lin^-^, CMP, and LSK populations (Fig 5I). In summary, these data demonstrate reduced GMP and marked inhibition of GMP maturation along the granulocytic lineage, with preservation and even increased monopoiesis upon deletion of the +37 kb *Cebpa* enhancer. Increased marrow erythropoiesis and blood platelets may represent redirection of CMP to MEP, and increased LSK and depletion of the LT-HSC and LSK/SLAM subsets may occur in response to GMP depletion.

### Effect of *Cebpa* Enhancer Deletion on Transcription Factor and Myeloid Cytokine Receptor Expression

**Fig 6:**
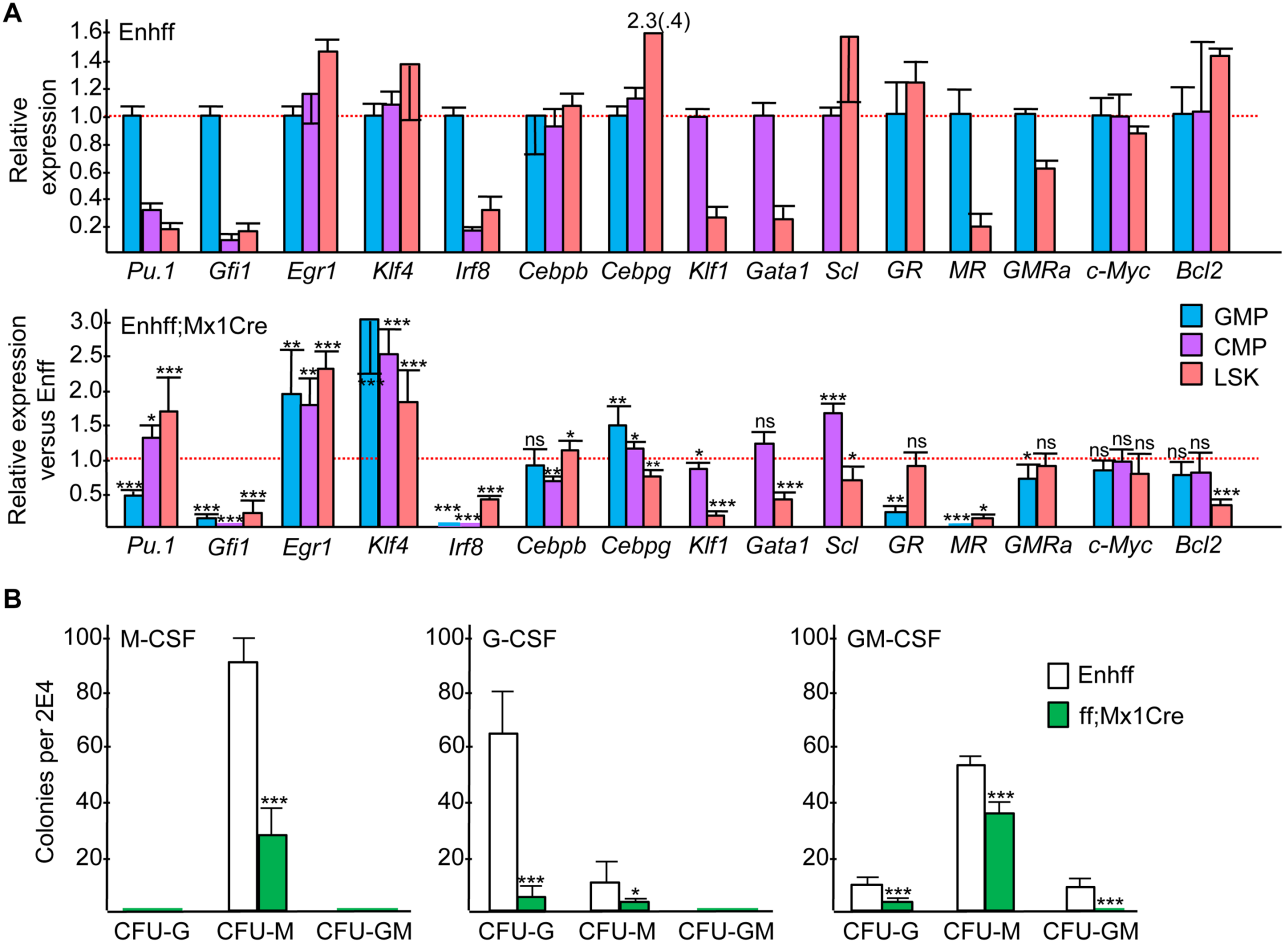
Effect of *in vivo* Enhancer Deletion on Selected Transcription Factor, Myeloid Cytokine Receptor, or Bcl2 Expression. **A**) Total cellular RNAs from the GMP, CMP, or LSK marrow subsets isolated from Enh(f/f) or Enh(f/f);Mx1-Cre mice that had been subjected to pIpC injections and allowed to recover for 4 wks were analyzed for relative expression of the indicated mRNAs (mean and SD from three determinations). Relative expression of each RNA is compared in GMP versus CMP versus LSK cells in Enh(f/f) marrow, with the average value in GMP or CMP set to 1.0 (top panel). Expression in Enh(f/f);Mx1-Cre divided by expression in Enh(f/f) marrow is shown for each RNA in each subset analyzed (bottom panel). Dashed red line is the 1.0 level for both graphs. GR—Gcsfr, MR—Mcsfr, GMRa—Gmcsfrα. **B**) Myeloid CFUs obtained from Enh(f/f) or Enh(f/f);Mx1-Cre mice exposed 4 wks earlier to pIpC were in enumerated after culture at 2E4 cells/mL in methylcellulose in the presence of M-CSF, G-CSF, or GM-CSF (mean and SD from six determinations).

The RNA samples used to evaluate *Cebpa* mRNA expression in GMP, CMP, or LSK marrow subsets from Enh(f/f) or Enh(f/f);Mx1-Cre mice exposed 4 wks earlier to pIpC were also evaluated for expression of several myeloid and erythroid transcription factors. Relative expression in GMP versus CMP versus LSK from Enh(f/f) mice and the ratio of expression in Enh(f/f);Mx1-Cre versus Enh(f/f) mice for each subset is shown (Fig 6A). *Pu*.*1* and *Gfi1* levels were increased in GMP compared with CMP or LSK. Enhancer deletion reduced *Pu*.*1* 2-fold in GMP, potentially reflecting direct regulation of *Pu*.*1* transcription by C/EBPα [20, 21], but increased *Pu*.*1* 1.3-fold in CMP and 3-fold in LSK. *Gfi1* was markedly reduced in GMP, CMP, and LSK, likely reflecting the role of Gfi-1 in mediating granulopoiesis [22], while *Egr1* and *Klf4* levels were increased in all three subsets, potentially reflecting their role in monopoiesis [23, 24]. *Irf8* levels were markedly reduced in GMP, CMP, and LSK cells in response to enhancer deletion despite the positive role Irf8 plays during monopoiesis [25] but consistent with 5-fold reduction in *Irf8* evident in the expanded monocyte progenitor population present in the marrow of *Cebpa* ORF(f/f);Mx1-Cre mice exposed to pIpC [26]. *Cebpb* levels were minimally affected by *Cebpa* enhancer deletion, of relevance given the ability of C/EBPβ to compensate for absence of C/EBPα during granulopoiesis [27, 28]. *Cebpg* levels were increased only 1.4-fold in GMP and 1.2-fold in CMP and were mildly reduced in LSK cells, of relevance given the 5-fold increase in *Cebpg* evident in LSK cells from adult mice lacking both copies of the *Cebpa* ORF following Mx1-Cre-mediated deletion [29] and given the adjacency of the *Cebpa* and *Cebpg* genes. Finally, RNAs encoding the erythroid factors *Gata1*, *Klf1*, and *Scl* were only minimally changed in CMP and reduced in LSK despite the increased erythropoiesis observed, perhaps reflecting a post-transcriptional effect of reduced C/EBPα on the activity of one or more of their cognate transcription factors.

In conducting myeloid CFU assays, we utilized IL-3, IL-6, and SCF due to the ability of this cytokine combination to support growth of CFU-G, CFU-M, and CFU-GM and due to markedly reduced *Gcsfr*, *Mcsfr*, and *Gmcsfrα* mRNA expression in the absence of C/EBPα [30, 31]. We evaluated the levels of these myeloid cytokine receptor mRNAs in GMP and LSK from Enh(f/f) versus Enh(f/f);Mx1-Cre mice 4 wks after pIpC exposure (Fig 6A). *Mcsfr* was reduced >12-fold by enhancer deletion in both subsets, *Gcsfr* was reduced 5-fold in GMP but was not affected in LSK, and *Gmcsfrα* was reduced only minimally in GMP or LSK. Reduced *Mcsfr* in GMP is evident despite increased CFU-M in IL-3/IL-6/SCF, whereas reduced *Gcsfr* in GMP could reflect markedly diminished CFU-G numbers. To evaluate the functional consequences of these changes in receptor expression, we conducted CFU assays in M-CSF, G-CSF, or GM-CSF (Fig 6B). CFU-M were reduced 3-fold in M-CSF, and CFU-G were reduced 11-fold in G-CSF, whereas CFU-G were only reduced 4-fold and CFU-M 1.5-fold in GM-CSF. Relative sparing of CFU-G and CFU-M in GM-CSF may reflect the ability of this cytokine to mediate emergency granulopoiesis in the absence of C/EBPα via induction of C/EBPβ [28, 32].

### Effect of *Cebpa* Enhancer Deletion on Marrow Progenitor Proliferation and Survival

**Fig 7:**
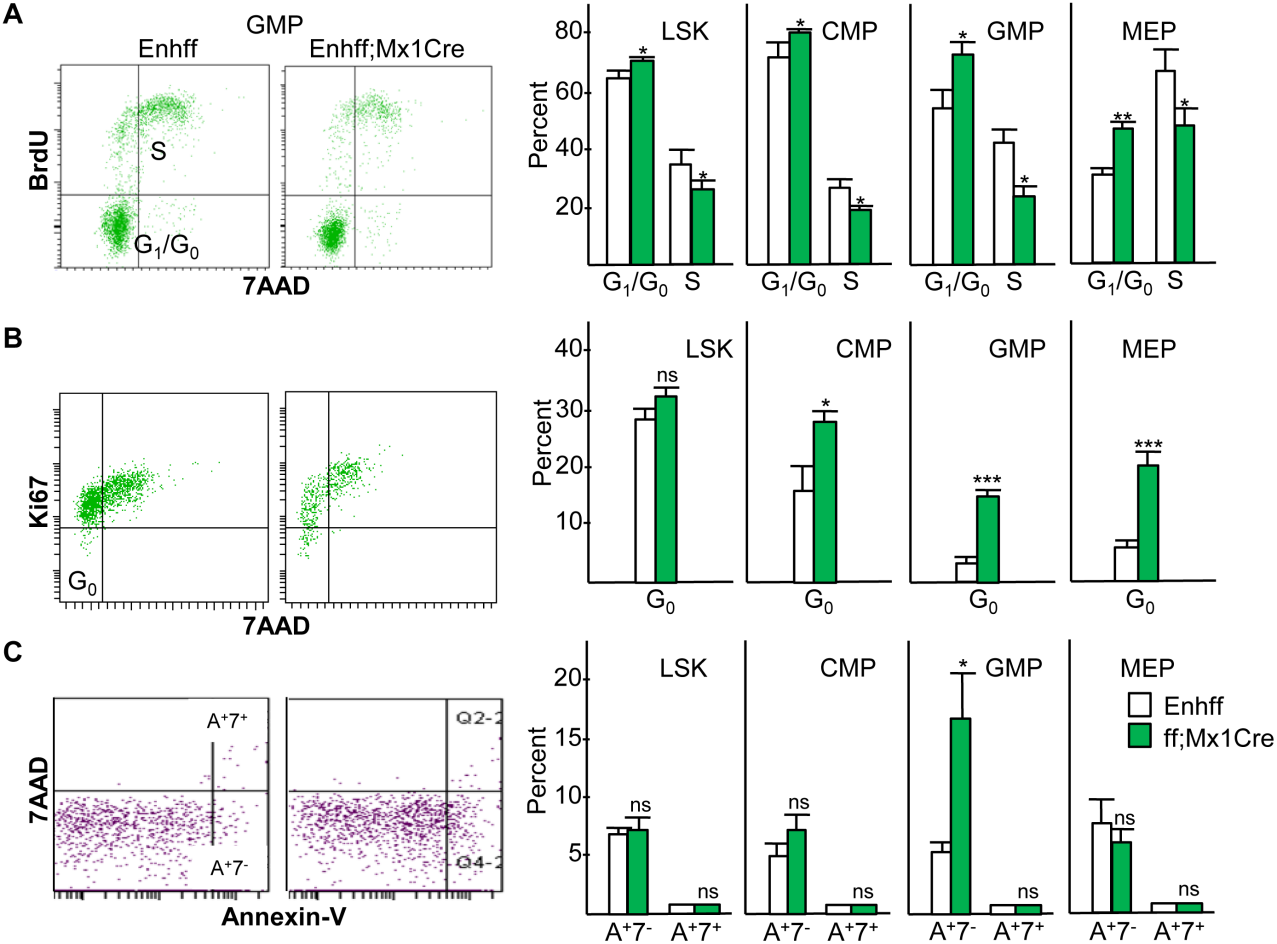
Effect of *in vivo* Enhancer Deletion on Progenitor Cell Proliferation and Survival. **A**) Enh(f/f) or Enh(f/f);Mx1-Cre mice exposed 4 wks earlier to pIpC received a BrdU injection 3 hr prior to marrow harvest, followed by staining for surface markers to allow gating on the LSK, CMP, GMP, and MEP subsets and for intracellular incorporation of BrdU and 7AAD into DNA after fixation, permeabilization, and DNase exposure. G_0_/G_1_ (BrdU^-^7AAD^-^) and S phase (BrdU^+^) cells were then enumerated (mean and SD from three determinations). **B**) Marrow from Enh(f/f) or Enh(f/f);Mx1-Cre mice exposed 4 wks earlier to pIpC were stained for LSK, CMP, GMP, and MEP and for intracellular Ki67 and 7AAD. G_0_ (Ki67^-^7AAD^-^) cells were enumerated (mean and SD from three determinations). **C**) Marrow from a similar group of mice was stained for the same progenitors, for surface Annexin-V, and for permeability to 7AAD in the absence of fixation. Non-viable Annexin-V^+^7AAD^+^ (A^+^7^+^) and early apoptotic Annexin-V^+^7AAD^-^ (A^+^7^-^) cells were then enumerated (mean and SD from three determinations).

As C/EBPα can directly inhibit cell cycle progression and apoptosis in myeloid cells [33–36], we evaluated the effect of *Cebpa* enhancer deletion on relevant parameters in the LSK, CMP, GMP, and MEP marrow subsets from Enh(f/f) versus Enh(f/f);Mx1-Cre mice 4 wks after completion of pIpC injections. BrdU/7AAD staining 3 hr after BrdU injection demonstrated increased G_0_/G_1_ and reduced S cell cycle phase cells in each subset in the absence of the +37 kb *Cebpa* enhancer, with the most evident effect in the GMP and MEP populations (Fig 7A). Of note, as expected the latter subsets had reduced G_0_/G_1_ and increased S phase cells compared with the earlier LSK or CMP subsets in Enh(f/f) mice. Similarly, Ki67/7AAD staining revealed reduced quiescent, G_0_ phase cells as Enh(f/f) LSK cells progress to CMP and then to GMP or MEP, and enhancer deletion increased the proportion of G_0_ cells in the CMP, GMP, and MEP subsets (Fig 7B). Thus, reduced GMP evident in the absence of the *Cebpa* enhancer may in part reflect reduced proliferation, whereas LSK expansion occurs despite their mildly reduced proliferative status. Of note, although C/EBPα inhibits E2F activation of the *c-Myc* promoter [37], *c-Myc* RNA expression in GMP, CMP, or LSK was unaffected by *Cebpa* enhancer deletion (Fig 6A). Exogenous *Cebpa* markedly reduces proliferation of marrow myeloid progenitors and additional cell types [33, 38]; therefore, reduced LSK, CMP, GMP, and MEP proliferation consequent to *Cebpa* enhancer deletion apparently reflects direct or indirect pathways connecting C/EBPα to cell cycle progression not previously evaluated.

Annexin-V/7AAD staining demonstrated only minimal numbers of Annexin-V^+^7AAD^+^ non-viable cells in the LSK, CMP, GMP, or MEP populations, with an increased number of Annexin-V^+^7AAD^-^ early apoptotic cells, from 5% to 16%, in response to enhancer deletion only in the GMP subset (Fig 7C). Increased apoptosis in the absence of the *Cebpa* enhancer may thus also contribute to reduced GMP numbers. Of note, despite the ability of C/EBPα to activate the *Bcl2* promoter in cooperation with NF-κB p50 [36], enhancer deletion did not affect *Bcl2* mRNA levels in GMP or CMP, though *Bcl2* was reduced 3-fold in the LSK subset (Fig 6A).

### Effect of *Cebpa* Enhancer Deletion on Functional Long-Term Hematopoietic Stem Cells

**Fig 8:**
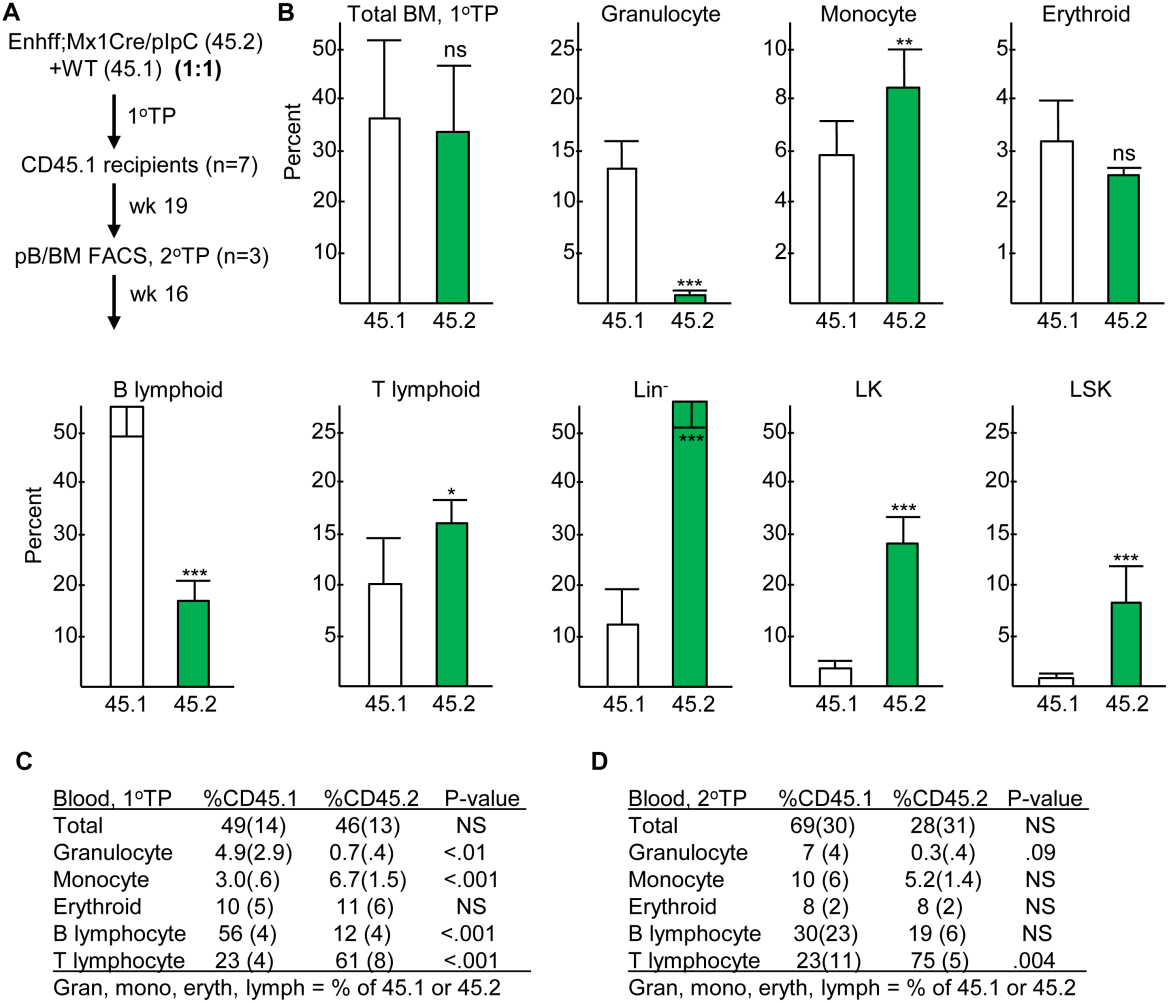
Effect of *in vivo* Enhancer Deletion on Functional Long-Term Hematopoietic Stem Cells. **A**) Diagram of competitive transplantation assay. 2E5 CD45.2^+^ nucleated marrow cells from Enh(f/f);Mx1-Cre mice exposed 4 wks earlier to pIpC were mixed with equal numbers of CD45.1^+^ WT marrow cells and transplanted into lethally irradiated WT recipients. At 19 wks, peripheral blood (pB) and bone marrow (BM) cells were analyzed, and 1E6 marrow cells were transplanted into secondary transplant (2°TP) recipients, 1 recipient/donor. Those surviving were then analyzed 16 wks later. **B**) Percent of CD45.1^+^ or CD45.2^+^ cells amongst total marrow nucleated cells, and the percent of CD11b^+^Gr-1^+^ granulocytes, CD11b^+^Gr-1^-^ monocytes, Ter119^+^ erythroblasts, B220^+^ B lymphoid cells, CD3^+^ T lymphoid cells, Lin^-^, Lin^-^Sca-1^-^c-kit^+^ (LK), or Lin^-^Sca-1^+^c-kit^+^ (LSK) cells within the CD45.1^+^ or CD45.2^+^ subsets. **C**) Percent of CD45.1^+^ or CD45.2^+^ cells amongst nucleated peripheral blood cells in primary transplant recipients (mean and SD; n = 7). **D**) Percent of CD45.1^+^ or CD45.2^+^ cells amongst nucleated peripheral blood cells in secondary transplant recipients (mean and SD; n = 3).

Functional LT-HSCs capable of long-term, multi-lineage hematopoietic reconstitution represent a small subset of the FACS-defined LSK/SLAM or LT-HSC subsets. To evaluate the consequence of *Cebpa* enhancer deletion for their frequency in adult marrow, equal numbers of CD45.2^+^ nucleated marrow cells isolated from Enh(f/f);Mx1-Cre mice exposed 4 wks earlier to pIpC and CD45.1^+^ cells from WT mice were transplanted into lethally irradiated CD45.1^+^ WT recipients. 19 wks later, at which point hematopoietic cells reflect output from functional LT-HSC, marrow and blood cells were analyzed for CD45.1 and CD45.2 expression, for lineage markers, and for Sca-1 and c-Kit expression (Fig 8A–8C). After primary transplantation, the proportion of CD45.1^+^ and CD45.2^+^ total nucleated cells in marrow or blood were equivalent, on average, indicating the presence of similar numbers of functional LT-HSC in the enhancer-deleted and WT marrow cells at the time of transplantation.

Granulocytes represented a much smaller fraction of the CD45.2^+^ subset in primary transplant recipients compared with the CD45.1^+^ subset, in marrow or blood, providing further evidence for a hematopoietic-intrinsic impairment in granulopoiesis consequent to *Cebpa* enhancer deletion. Increased monocytes and reduced B cells in both marrow and blood and markedly expanded Lin^-^, LK, and LSK marrow populations in the CD45.2^+^ compared with the CD45.1^+^ population also confirmed the marrow-intrinsic nature of these changes when assessed in WT recipients. Increased marrow and blood T cells were also evident in the CD45.2^+^ versus CD45.1^+^ subsets.

Marrow from primary transplant recipients was transplanted into lethally irradiated CD45.1^+^ WT secondary recipients. Among those mice that survived until 16 wks post-secondary transplantation, the proportion of total CD45.2^+^ and CD45.1^+^ cells were not statistically different, with retention of multi-lineage CD45.2^+^ cell engraftment, consistent with absence of a deficiency in functional LT-HSC in the initial Enh(f/f);Mx1-Cre graft (Fig 8D). There was again a trend towards reduced granulocytes in the CD45.2^+^ subset, but this did not reach statistical significance. Early death of several secondary recipients may reflect their increased average CD45.2^+^ proportion and so reduced total granulopoiesis compared to primary transplant recipients, predisposing to septic death in the setting of marrow transplantation where donor radiation weakens intestinal mucosa integrity facilitating bacterial entry into the bloodstream.

## Discussion

The main conclusion of this study is that the +37 kb *Cebpa* enhancer is a critical, hematopoietic-specific regulator of *Cebpa* transcription. In addition, availability of adult mice lacking the *Cebpa* enhancer provided a hypomorphic model that could be evaluated to gain new insight into regulation of hematopoiesis by C/EBPα. Consistent with results obtained with mice lacking both copies of the C/EBPα ORF, deletion of the *Cebpa* enhancer led to marked neutropenia, reduced GMP, expanded LSK, and increased erythroid progenitors. Enh(f/f);Mx1-Cre mice exposed to pIpC also manifested monocytosis, impaired B lymphopoiesis, and functional LT-HSC retention.

The *Cebpa* +37 kb enhancer and the -725/+125 bp *Cebpa* promoter are sufficient to direct hCD4 transgene expression to marrow GMP, CMP, LSK, and CLP, and to functional LT-HSC, with little expression in non-hematopoietic tissues [8]. Point mutation of seven Ets sites in the *Cebpa* enhancer in the 32Dcl3 myeloid line markedly reduces *Cebpa* mRNA expression [11]. We now find that Cre-mediated enhancer deletion in myeloid progenitors *in vitro*, or Mx1-Cre, Vav-Cre, or CMV-Cre mediated enhancer deletion *in vivo* also markedly reduces *Cebpa* mRNA expression, with 14-fold reduction in GMP *Cebpa* mRNA in response to Mx1-Cre. In contrast, germline enhancer deletion by CMV-Cre did not alter *Cebpa* expression in adipocytes, liver, lung, small intestine, skeletal muscle, or kidney. Together, the transgenic and enhancer deletion data indicate that the +37 kb *Cebpa* enhancer is necessary and sufficient for directing hematopoietic *Cebpa* gene transcription, with high-level *Cebpa* expression in other lineages, e.g. adipocytes, hepatocytes, or type II pneumocytes, dependent on other regulatory elements within the *Cebpa* locus.

The *Cebpa* gene is flanked by the *Cebpg* gene 64 kb upstream and by the *Slc7a10* gene, encoding an amino acid transport protein expressed only in neurons, 68 kb downstream. *Cebpa* enhancer deletion did not reduce *Cebpg* expression in marrow GMP, CMP, or LSK, suggesting a barrier to communication between the +37 kb *Cebpa* enhancer and the -64 kb *Cebpg* promoter.

*Cebpa* ORF(-/-), germline-deleted mice manifest neonatal lethality due to hepatic defects, with marked neutropenia and monocytopenia [19, 30], and *Cebpa* ORF(-/-) fetal liver cells are also deficient in generating neutrophils and monocytes and manifest increased erythropoiesis [31, 39]. *Cebpa* ORF(f/f);Mx1-Cre mice exposed to pIpC have markedly reduced blood neutrophils, monocytes, and eosinophils, with increased platelets, 18-fold reduced marrow GMP, 5-fold increased CMP, 4-fold increased MEP, and 32-fold increased LSK cells [3]. *Cebpa* Enh(f/f);Mx1-Cre mice develop related changes, with neutropenia, reduced GMP, LSK expansion, and increased BFU-E. However, the degree of GMP and neutrophil reduction and LSK expansion was less, eosinophils were retained, and marrow CFU-M are increased rather than absent. These differences in myelopoiesis likely reflect the effect of residual, albeit low-level *Cebpa* in enhancer-deleted GMP and supports our prior observations with *Cebpa* shRNA-transduced myeloid progenitors [4]. In the latter study, 3-fold *Cebpa* knockdown impaired granulopoiesis while increasing monopoiesis, whereas 6-fold *Cebpa* knockdown prevented commitment to either lineage, increased BFU-E formation even in the absence of EPO, and enabled morphologic blast accumulation with indefinite, cytokine-dependent myeloid CFU replating, the latter also seen in the current study upon *Cebpa* enhancer deletion. High level C/EBPα, as seen in CFU-G, may homodimerize to direct granulopoiesis, whereas reduced C/EBPα, as seen in CFU-M, may heterodimerize with AP-1 proteins via their leucine zipper domains to mediate monopoiesis [4, 40, 41]. Homozygous enhancer-deleted (ΔΔ) fetal liver cells also had markedly reduced granulocytes, though monocytes were retained. Interestingly, heterozygous enhancer-deleted (Δ+) fetal liver had 5-fold reduced granulocytes whereas adult Δ+ marrow neutrophils were not reduced, suggesting greater sensitivity of fetal liver granulopoiesis to reduced C/EBPα. Earlier work similarly revealed a >2-fold reduction in fetal liver granulocytes in *Cebpa* ORF (+/-) embryos [39].

The *Cebpa* enhancer has increasing H3K4me1 and K3K27Ac activating histone modifications as LT-HSC mature to ST-HSC, MPP, CMP, and finally GMP, and while these marks are minimal in MEP they are readily evident also in CLP, albeit at reduced levels compared with GMP [11]. Potentially related to the apparent activity of the enhancer in at least a subset of the CLP population, the *Cebpa* enhancer/promoter directs high-level hCD4 transgene expression to 36% of CLP. In addition, sorting of marrow from these transgenic mice into hCD4^-^ and hCD4^+^ fractions followed by plating in methylcellulose with IL-7 identified B cell/macrophage (B/Mo) CFUs exclusively in the hCD4^+^ population, with strikingly increased surface c-kit expression on the CD19^+^ B cells from these colonies, a marker of immaturity [8]. Although B cells were not reduced in the marrow of Enh(f/f);Mx1-Cre mice after pIpC exposure, these earlier observations and the findings that *Cebpa* enhancer deletion reduces the number of B lymphoid CFUs obtained in IL-7 and reduces Enh(f/f);Mx1-Cre-derived B cells after transplantation into WT recipients suggests that *Cebpa* is required at one or more step in early B cell development, including perhaps formation of B/Mo bi-potent progenitors. Alternatively, reduced B lymphopoiesis upon *Cebpa* enhancer deletion may reflect redirection of lymphoid-myeloid (LMP) progenitors to GMP and away from CLP. Of note, cells with B/Mo potential have been detected in marrow, although there *in vivo* relevance is uncertain [42–44], and exogenous C/EBPα converts B cells to macrophages [45]. Further insight into the role of C/EBPα in B cell development might be gained by deleting the +37 kb *Cebpa* enhancer specifically in the B lineage, as we will pursue in future studies.

**Fig 9:**
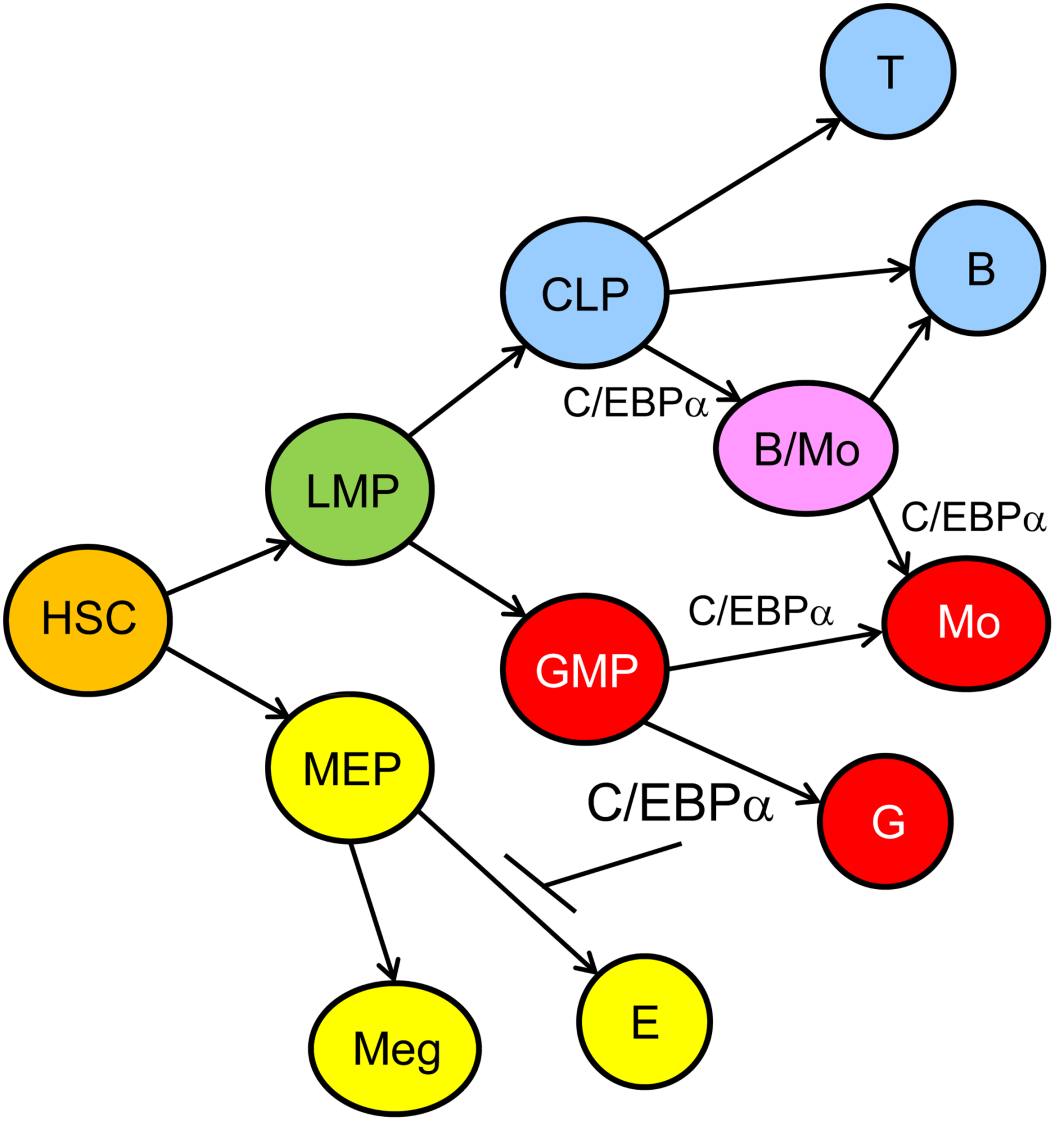
Model for the Role of C/EBPα During Hematopoiesis. In this model, HSC give rise to lymphoid-myeloid progenitors (LMP) and to MEP, MEP generate megakaryocyte (Meg) and erythroid (E) progenitors, and GMP give rise to granulocytic progenitors (G) in the presence of high-level C/EBPα and to monocytic progenitors (Mo) in the presence of low-level C/EBPα. CLP give rise to T and B cell progenitors in the absence and to bipotent B/Mo progenitors in the presence of C/EBPα. The latter diverge to B cell or monocytic progenitors, again correlated with and potentially guided by loss or retention of C/EBPα.

A model of hematopoietic lineage determination focused on the role of C/EBPα based on our findings and those of others, is shown (Fig 9). In this model high-level C/EBPα is required for granulopoiesis and lower level for monopoiesis, high-level C/EBPα inhibits erythropoiesis, and C/EBPα contributes to formation of a B/Mo progenitor arising from CLP.

Several prior studies addressed the effect of bialleleic *Cebpa* ORF deletion on functional LT-HSC in adult marrow, two finding increased LT-HSC after primary transplantation and one demonstrating impairment after both primary and secondary transplantation [3, 14, 15]. In the current study, functional LT-HSC were preserved after *Cebpa* enhancer deletion, perhaps reflecting the <2-fold reduction of *Cebpa* mRNA observed in the LSK/SLAM population, which might mirror effects on *Cebpa* expression in functional LT-HSC. The ability of the *Cebpa* enhancer and promoter to nevertheless direct hCD4 transgene expression to long-term repopulating LT-HSC might either indicate that in this population another *Cebpa* regulatory element besides the +37 kb enhancer is also sufficient in this regard or that another element suppresses *Cebpa* transcription in functional LT-HSC. Of note, FACS-defined LSK/SLAM cells are markedly depleted in the marrow of Enh(f/f);Mx1-Cre mice exposed to pIpC. This may represent depletion of the majority of this population, other than the more quiescent, functional LT-HSC, to enable LSK expansion in response to reduced myeloid and B lineage progenitors.

Exogenous C/EBPα inhibits G1 to S cell cycle progression in multiple cell types via several mechanisms, including via direct interaction with E2F1 in 32Dcl3 myeloid cells [35]. However, *Cebpa* ORF deletion did not alter BrdU incorporation into marrow LSK cells, and *Cebpa* shRNA knockdown did not alter myeloid progenitor cell cycle parameters *in vitro* [3, 4]. Similarly, we now find that reduced *Cebpa* consequent to enhancer deletion has minimal effect on proliferation of the LSK or CMP marrow subsets or *c-Myc* levels while reducing G1 to S cell cycle progression in GMP. Thus, the role of C/EBPα in regulation of cell cycle progression may depend on its level of expression, with high levels markedly slowing and low levels mildly slowing proliferation.

Exogenous C/EBPα inhibits apoptosis consequent to cytokine withdrawal from hematopoietic cells, in part via Bcl-2 induction in cooperation with NF-κB p50 [36, 46]. Consistent with these findings, *Cebpa* enhancer deletion increases early apoptosis in GMP, perhaps together with slowed proliferation contributing to their reduced numbers.

Multiple mechanisms lead to diminished but generally not absent C/EBPα expression or activity during the pathogenesis of acute myeloid leukemia, including alterations leading to reduced transcription, reduced translation, or reduced protein activity or stability [5]. Impaired but not absent myelopoiesis due to +37 kb *Cebpa* enhancer deletion, with long-term, cytokine-dependent myeloid CFU replating, may represent preleukemic phenotypes absent in mice completely lacking C/EBPα. Of note, Bcr-abl expression in *Cebpa* ORF(-/-) hematopoietic cells generates erythroleukemia rather than myeloid leukemia [47], likely reflecting the need for a minimal number of GMP to act as substrates for myeloid transformation [48, 49]. As we have not observed signs of leukemic transformation in a cohort of enhancer-deleted mice over a 55 wk period, expression of additional proliferative oncoproteins may be required, as we will pursue in future studies. Finally, as we discussed previously [11], sequencing of 110 human AML cases did not reveal point mutations or small insertions/deletions within the homologous +42 kb *CEBPA* enhancer [50]. This may reflect the fact that heterozygous absence of the +37 kb enhancer in adult mice does not significantly alter myelopoiesis and the greater efficiency of altering pathways that regulate the enhancer. For example, Runx1 binds and activates the +37 kb *Cebpa* enhancer [7], and ChIP-Seq demonstrated that the RUNX1-ETO AML oncoprotein binds specifically at the +42 kb *CEBPA* enhancer, but not the *CEBPA* promoter, in two patient samples and in the Kasumi-1 cell line [51], likely leading to *Cebpa* trans-repression. In addition, our finding that Pu.1 and C/EBPα also bind and activate the enhancer via conserved *cis* elements [11] suggests that alterations that reduce the expression or activity of either of these transcription factors might reduce *CEBPA* transcription to further contribute to myeloid transformation.
